# Supplementary material for: Early Enteral Nutrition (within 48 h) for Patients with Sepsis or Septic Shock: A Systematic Review and Meta-Analysis
Source: Nutrients. 2024 May 22;16(11):1560. doi: 10.3390/nu16111560 (PMC11174497; doi:10.3390/nu16111560)
Supplement: Supplementary file 1 [file nutrients-16-01560-s001.zip › nutrients-2986879-supplementary.pdf]

EARLY ENTERAL NUTRITION (WITHIN 48 HOURS) FOR PATIENTS WITH SEPSIS OR SEPTIC SHOCK: A SYSTEMATIC REVIEW AND META-ANALYSIS

SUPPLEMENTAL DIGITAL CONTENT

Figure Legend

Figure S1. Funnel plot for EEN versus DEN. Outcome: 28-days mortality.

Figure S2. Funnel plot for EEN versus DEN. Outcome: Illeus.

Figure S3. Funnel plot for EEN versus DEN. Outcome: Ventilator-associated pneumonia.

Table Legend

Table S1. Literature Search

Table S2. Summary characteristics of included, and excluded studies.

Table S3. Summary of findings table for RCTs.

Table S4. Summary of findings table for NRS

Figure S1. Funnel plot for EEN versus DEN. Outcome: 28-days mortality.

Study or Subgroup	Early enteral nutrition Events	Early enteral nutrition Total	Delayed enteral nutrition Events	Delayed enteral nutrition Total	Weight	Odds Ratio M–H, Fixed, 95% CI
**1.7.1 RCTs**						
Beale 2008	12	27	10	28	52.3%	1.44 [0.49, 4.25]
Sun 2019	4	26	6	27	47.7%	0.64 [0.16, 2.58]
**Subtotal (95% CI)**		**53**		**55**	**100.0%**	**1.06 [0.45, 2.46]**
Total events	16		16			
Heterogeneity: $Chi^2 = 0.82$, df = 1 (P = 0.37); $I^2 = 0\%$						
Test for overall effect: Z = 0.13 (P = 0.90)						
**1.7.2 NRS**						
Jiang 2020	24	85	34	78	11.0%	0.51 [0.27, 0.98]
Liu 2018	13	35	12	28	3.6%	0.79 [0.29, 2.17]
Ortiz–Reyes 2022	159	526	29	100	14.7%	1.06 [0.66, 1.70]
Sun 2017	5	46	8	36	3.5%	0.43 [0.13, 1.44]
Wang 2023	196	818	204	818	67.2%	0.95 [0.76, 1.19]
**Subtotal (95% CI)**		**1510**		**1060**	**100.0%**	**0.89 [0.74, 1.08]**
Total events	397		287			
Heterogeneity: $Chi^2 = 5.13$, df = 4 (P = 0.27); $I^2 = 22\%$						
Test for overall effect: Z = 1.19 (P = 0.24)						

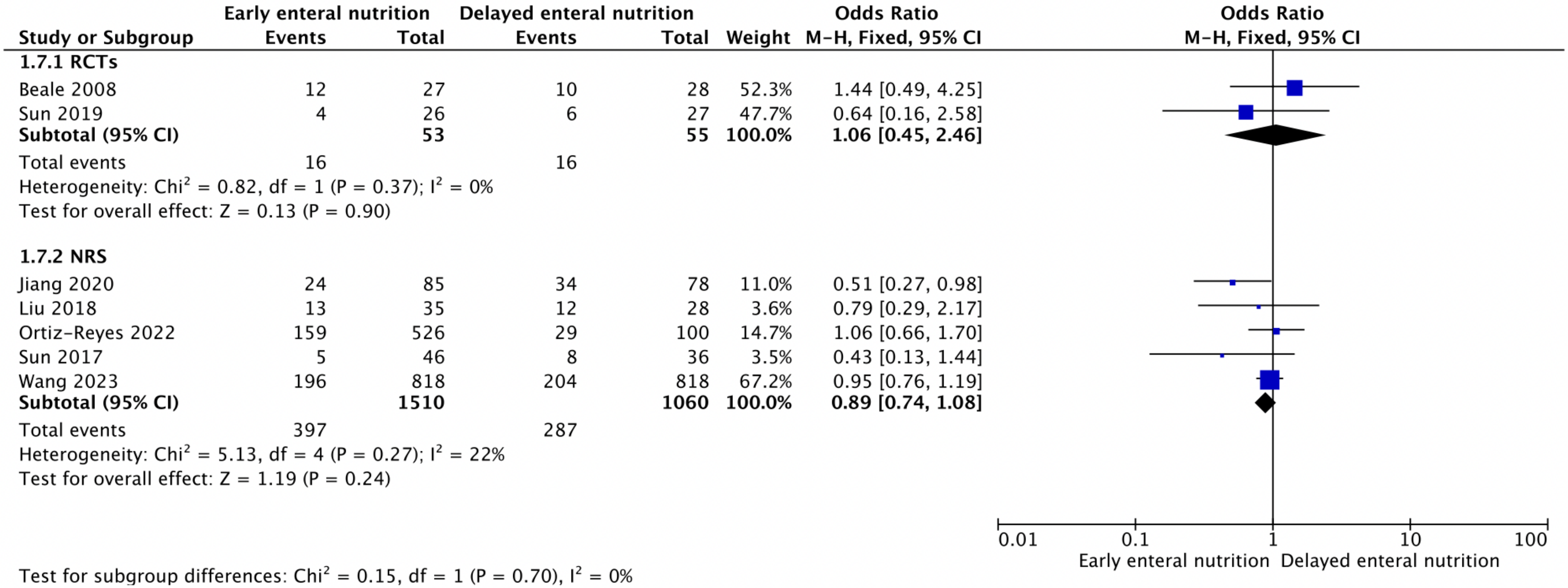

Test for subgroup differences: $Chi^2 = 0.15$, df = 1 (P = 0.70), $I^2 = 0\%$

Figure S2. Funnel plot for EEN versus DEN. Outcome: Illeus.

Study or Subgroup	Early enteral nutrition Events	Early enteral nutrition Total	Delayed enteral nutrition Events	Delayed enteral nutrition Total	Weight	Odds Ratio M-H, Fixed, 95% CI
**1.5.1 RCTs**						
Patel 2019	0	15	0	16		Not estimable
**Subtotal (95% CI)**		**15**		**16**		**Not estimable**
Total events	0		0			
Heterogeneity: Not applicable						
Test for overall effect: Not applicable						
**1.5.2 NRS**						
Patel 2014a	1	37	0	7	40.2%	0.62 [0.02, 16.64]
Patel 2014b	1	14	1	8	59.8%	0.54 [0.03, 9.99]
**Subtotal (95% CI)**		**51**		**15**	**100.0%**	**0.57 [0.06, 5.04]**
Total events	2		1			
Heterogeneity: $Chi^2 = 0.00$, df = 1 (P = 0.95); $I^2 = 0\%$						
Test for overall effect: Z = 0.51 (P = 0.61)						

Test for subgroup differences: Not applicable

Odds Ratio
M-H, Fixed, 95% CI

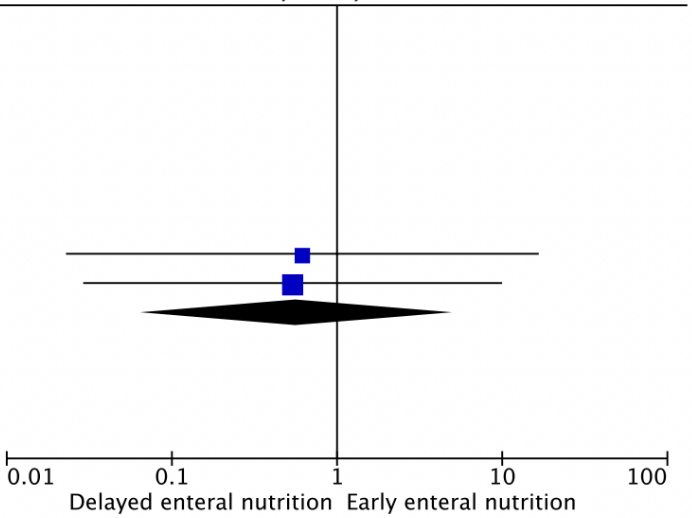

Figure S3. Funnel plot for EEN versus DEN. Outcome: Ventilator-associated pneumonia.

Study or Subgroup	Early enteral nutrition Events	Early enteral nutrition Total	Delayed enteral nutrition Events	Delayed enteral nutrition Total	Weight	Odds Ratio M-H, Fixed, 95% CI
**1.6.1 RCTs**						
Patel 2019	0	15	1	16	100.0%	0.33 [0.01, 8.83]
**Subtotal (95% CI)**		**15**		**16**	**100.0%**	**0.33 [0.01, 8.83]**
Total events	0		1			
Heterogeneity: Not applicable						
Test for overall effect: Z = 0.66 (P = 0.51)						
**1.6.2 NRS**						
**Subtotal (95% CI)**		**0**		**0**		**Not estimable**
Total events	0		0			
Heterogeneity: Not applicable						
Test for overall effect: Not applicable						
**Total (95% CI)**		**15**		**16**	**100.0%**	**0.33 [0.01, 8.83]**
Total events	0		1			
Heterogeneity: Not applicable						
Test for overall effect: Z = 0.66 (P = 0.51)						
Test for subgroup differences: Not applicable						

Odds Ratio
M–H, Fixed, 95% CI

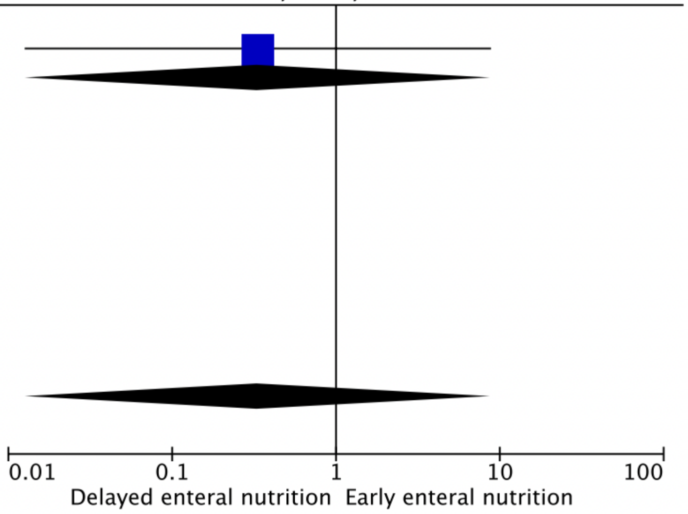

Table S1. Literature Search

Search #1	
**Type of search**	New
**Database**	▪ MEDLINE<br>▪ MEDLINE(R) Epub Ahead of Print<br>▪ MEDLINE(R) Daily Update
**Platform**	Ovid
**Search Date**	June 30 of 2023
**Search Date Range**	None
**Language restriction**	None
**Other limits**	None
**Search strategy**	1. (early or premature).tw. (1946067)<br>2. exp Enteral Nutrition/ (21915)<br>3. ((enteral or enteric) adj5 (nutrition or feeding)).tw. (16605)<br>4. (force adj3 feeding*).tw. (498)<br>5. (tube* adj3 feeding).tw. (8851)<br>6. (intragastric adj3 feeding).tw. (322)<br>7. (intestinal adj3 feeding).tw. (550)<br>8. (intraintestinal adj3 feeding).tw. (9)<br>9. PEG line*.tw. (12)<br>10. en.tw. (108464)<br>11. (feeding adj5 therap*).tw. (1205)<br>12. or/2-11 (141187)<br>13. 1 and 12 (15392)<br>14. exp Sepsis/ (141246)<br>15. exp Shock, Septic/ (24940)<br>16. sepsis.tw. (117459)<br>17. (bloodstream adj2 infection*).tw. (13417)

	18. (py?emi* or pyohemia*).tw. (262)<br>19. septic*.tw. (85482)<br>20. (blood adj2 poisoning).tw. (189)<br>21. (toxic adj2 shock).tw. (5057)<br>22. (endotoxi* adj2 shock*).tw. (4289)<br>23. or/14-22 (257845)<br>24. 24 13 and 23 (776)
**# of references Identified**	776
**# of references after removing duplicates**	416

**Search #2**	
**Type of search**	New
**Database**	▪ EMBASE
**Platform**	Embase.com
**Search Date**	June 30 of 2023
**Search Date Range**	None
**Language restriction**	None
**Other limits**	None
**Search strategy**	1. early:ti,ab OR premature:ti,ab (2673101)<br>2. 'enteric feeding'/exp (40191)<br>3. ((enteral OR enteric) NEAR/5 (nutrition OR feeding)):ti,ab (27029)<br>4. (force NEAR/3 feeding*):ti,ab (556)<br>5. (tube* NEAR/3 feeding):ti,ab (14076)<br>6. (intragastric NEAR/3 feeding):ti,ab (426)<br>7. (intestinal NEAR/3 feeding):ti,ab (699)<br>8. (intraintestinal NEAR/3 feeding):ti,ab (12)<br>9. peg AND line*:ti,ab (7285)<br>10. en:ti,ab (118495)<br>11. (feeding NEAR/5 therap*):ti,ab (1630)

	12. #2 OR #3 OR #4 OR #5 OR #6 OR #7 OR #8 OR #9 OR #10 OR #11 (180086)<br>13. #1 AND #12 (21203)<br>14. 'sepsis'/exp (340275)<br>15. 'septic shock'/exp (71701)<br>16. sepsis:ti,ab (184257)<br>17. (bloodstream NEAR/2 infection*):ti,ab (18902)<br>18. py?emi*:ti,ab OR pyohemia*:ti,ab (90)<br>19. septic*:ti,ab (121777)<br>20. (blood NEAR/2 poisoning):ti,ab (233)<br>21. (toxic NEAR/2 shock):ti,ab (6024)<br>22. (endotoxi* NEAR/2 shock*):ti,ab (5094)<br>23. #14 OR #15 OR #16 OR #17 OR #18 OR #19 OR #20 OR #21 OR #22 (432648)<br>24. #13 AND #23 (1640)<br>25. #13 AND #23 AND [embase]/lim (1512)
**# of references identified**	1512
**# of references identified after removing duplicates**	1694

**Search #3**	
**Type of search**	New
**Datebase**	▪ Cochrane Controlled Register of Trials (CENTRAL)
**Platform**	Ovid
**Search Date**	June 30 of 2023
**Search Date Range**	None
**Language restriction**	None
**Other limits**	None
**Search strategy**	1. (early or premature).tw. (148892)

	2. exp Enteral Nutrition/ (2119)<br>3. ((enteral or enteric) adj5 (nutrition or feeding)).tw. (5059)<br>4. (force adj3 feeding*).tw. (4)<br>5. (tube* adj3 feeding).tw. (1649)<br>6. (intragastric adj3 feeding).tw. (22)<br>7. (intestinal adj3 feeding).tw. (81)<br>8. (intraintestinal adj3 feeding).tw. (0)<br>9. PEG line*.tw. (0)<br>10. en.tw. (8143)<br>11. (feeding adj5 therap*).tw. (216)<br>12. or/2-11 (14581)<br>13. 1 and 12 (2483)<br>14. exp Sepsis/ (6594)<br>15. exp Shock, Septic/ (1236)<br>16. sepsis.tw. (10688)<br>17. (bloodstream adj2 infection*).tw. (1035)<br>18. (py?emi* or pyohemia*).tw. (8)<br>19. septic*.tw. (5661)<br>20. (blood adj2 poisoning).tw. (26)<br>21. (toxic adj2 shock).tw. (55)<br>22. (endotoxi* adj2 shock*).tw. (22)<br>23. or/14-22 (17299)<br>24. 24 13 and 23 (235)
**# of references identified**	235
**# of references identified after removing duplicates**	104

Table S2. Summary characteristics of included and excluded studies.

Author, year	Patel 2019
Country	United States
Study design	• Phase 3 single center pilot parallel group randomized (1:1) controlled pilot study
Configuration	• Was conducted in the medical ICU at Froedtert and the Medical college of wisconsin<br>• Registered with www.clinicaltrials.gov, number NCT 02025127<br>• Concealed allocation: 1:1 ratio for early trophic EN or no EN sealed envelopes labeled 'feed' or 'no feed'' determined by randomization<br>• Simple blinding<br>• Analyses were performed on an intention to treat basis<br>• Study arm protocol compliance achieved in 100 % of patients in the arly trophic EN and 94% in the "no EN" group
Population	• Mechanically ventilated ICU patients<br>• Inclusion criteria<br>○ Adults who where at least 18 years of age<br>○ Admitted to the medical ICU with primary diagnosis of septic shock<br>○ Mechanically ventilated within 24 hours of ICU admission<br>○ Septic shock<br>• Exclusion criteria<br>○ Do not resuscitate order<br>○ Major gastrointestinal bleeding<br>○ Any bowel surgery in the previous 30 days<br>○ Small bowel ischemia or obstruction<br>○ Protracted vomiting or ileus<br>○ Contraindication for enteral feeding tube replacement<br>○ Moribunfd as deemed by the treating physician<br>• Characteristics<br>○ Mean age (60 years)<br>○ Male number (18)

	○ Median BMI (22-35 Kg/m2)<br>○ Norepinephrine dose (0.08 ug/kg/min)<br>○ Combination vasopressors (29 %)<br>○ SOFA score (8-20)<br>○ Source of sepsis<br>■ Pulmonary (7)<br>■ Unknown (12)
Intervention	● Early trophic EN, limited to 600 Kcal/day, initiated within 24 hours of randomization during vasopressor use. Upon 3 hour discontinuation, EN increased by 20 ml every 4 hours to goal rate<br>● Protein goals were set at 1.2-2 g/kg of actual body weight per day<br>● Metoproclamide used for high GRV (>500 ml); glycemic control <180 mg/dl<br>● Institution uses low tidal volume ventilation (6 ml/kg) and timely antimicrobial treatment for sepsis
Control	● No enteral nutrition<br>● In no enteral nutrition group, enteral nutrition withheld until 3 hour post vasopressor discontinuation. Enteral nutrition started at 20 ml/h, increased by 20 ml every 4 hours to goal rate. Physicians encouraged 48 hour EN delay
Outcomes	● Ventilator free days (24-26 days)<br>● Hospital mortality (26%)<br>● Need for new renal replacement therapy (19 %)<br>● Change in severity of illness, defined by a change in 48 hour sequential organ failure assessment (SOFA) score (0(-7))

[redacted]

- Complications
    - Vomiting in the first 72 hours (32 %)
    - Ileus any day (of first 7) (0%)
    - Intestinal ischemia (0%)
    - Small bowel obstruction (0%)
    - VAP (3,2 %)

- Enrollment: 31 patients at 0.7 /month; 15 early enteral nutrition, 16 no enteral nutrition

- Early enteral nutrition group: 27 VFDs (IQR 24-28) vs no enteral group: 14 (IQR 0-22), p= 0.09. Hospital mortality: Early enteral nutrition 13 % vs no enteral nutrition 33, p= 0.22, New RRT: Early enteral nutrition 2 vs no enteral nutrition 4p= 0.654

Author, year	Beale 2008
Country	United Kingdom, Switzerland, Germany
Study design	• Randomized, controlled, double blind, single center study
Configuration	• Performed in the adult general ICU on Guys and St Thomas Hospital, London<br>• Written consent was obtained from each patient
Population	• Inclusion criteria<br>○ Age of 18 or older<br>○ Patients with potential infection and systemic inflammatory response<br>○ Organ dysfunction, such as pulmonary issues or metabolic acidosis<br>○ Oliguria<br>○ Thrombocytopenia<br>○ Hypotension<br>○ APACHE II score of at least 10 and a precipitating injury within 24 hours of ICU entry<br>○ ICU should be over three days, with likely need for enteral nutrition at least five days<br>• Exclusion criteria<br>○ Cardiogenic shock or severe congestive heart failure<br>○ Severe, preexisting, parenchymal liver disease with clinically significant portal hypertension (Childs C)<br>○ Documented chronic obstructive pulmonary disease<br>○ Pregnancy<br>○ AIDS<br>○ Immunosuppression<br>■ Chronic treatment using high dose steroids<br>■ Active radiotherapy or chemotherapy<br>■ Lymphoma<br>■ Cellular immune deficiency<br>• Characteristics<br>○ Mean age 60 years<br>○ Mean BMI 25<br>○ SOFA score on day 0<br>■ Mean 7<br>○ Requiring mechanical ventilation 98%<br>○ Patients with sepsis 100%
Intervention	• Enteral pharmaco nutrition supplement (Intestamin)

	● Within 24 hours after enrollment at 21 ml/hr via nasogastric tube, for up to 10 days<br>● Intestamina received immininutrient containing Recovan<br>● To offset any initial caloric deficit to allow tight blood sugar control, patients received intravenous 20 % glucose until their feeding target was met<br>● Of 55 patients who underwent randomization, 27 received intestamine/recovan
Control	● Control supplement<br>● From day 2 patients also received a complete enteral formula<br>● Control supplement received Fresubin original<br>● Of 55 patients who underwent randomization, 28 received control feeds
Outcomes	● SOFA and APACHE II scores were assessed on the day of admission<br>● The 28 day mortality and 6 month mortality were assessed by separate follow up<br>● ICU/hospital mortality<br>○ Assessed at discharge<br>● Gastrointestinal tolerance was assessed daily<br>● Required renal replacement therapy 60%<br>● Diarrhea was higher in the intervention group than the control group<br>● The study found no significant discrepancies in mortality or length of stay at various time points between the two groups. Initial organ dysfunction variations were observed but normalized, with a quicker improvement in daily total SOFAscore over ten days<br>● Total number of respiratory infection<br>○ Intervention 13, control 9<br>● Hospital mortality<br>○ Intervention 25%, control 26%<br>● 28 day mortality<br>○ Intervention 18 %, control 11%

Author, year	Haac 2018
Country	United States
Study design	● Retrospective cohort
Configuration	● This study was reviewed and approved by the University of Maryland Institutional Review Board

Population	● Inclusion criteria<br>○ Adult patients aged 18 years and older, admitted to the ICU for a minimum of 72 hours with soft tissue disease<br>● Exclusion criteria<br>○ Patients with disseminated infections<br>○ Polymicrobial soft tissue infections<br>○ Anaerobic wound cultures<br>○ Specific aerobic organisms such as methicillin-resistant Staphylococcus aureus, methicillin-sensitive S. aureus, and Escherichia coli.<br>● Characterization<br>○ Septic on presentation 72%<br>○ Disseminated infection 26%<br>○ BMI 37<br>○ Diabetes 57%<br>○ Current smoker 24%<br>○ Alcohol abuse 15%<br>○ Respiratory disease 22%<br>○ CHF 15%<br>○ Male 60%<br>○ Age in years (mean) 59<br>○ SOFA score (mean) 7.4<br>○ Anatomic location of infection<br>■ Extremity<br>● EEN 77%<br>● DEN 58%<br>■ Perineum<br>● EEN 41%<br>● DEN 50%<br>■ Abdomen/retroperitoneum<br>● EEN 41%<br>● DEN 14%<br>■ Immunosupresed<br>● EEN 11%<br>● DEN 0%<br>■ Alcohol abuse<br>● EEN 14%

	● DEN 19%<br>■ Septic<br>● EEN 7.5%<br>● DEN 7.2%<br>■ Vaso actives use<br>● ENN 59%<br>● DEN 53%
Intervention	● Half of the patients were started on enteral nutrition within 24 hours<br>● Delays were attributed to clinical factors: severe sepsis/vasoactive needs, intubated patients without GI access, ileus, uncontrolled GI leakage, GI bleed concerns, and colonic diversion anticipation. Tube feeding used, except for one patient with an immune-enhanced formula. No early total parenteral nutrition (TPN)
Control	● Eleven of 16 patients who had delay in receiving nutrition
Outcomes	● SOFA scores were calculated using data from the first 24 hours of ICU admission<br>● The text concerns the assessment of hospital-acquired infections, including diagnosis, treatment, surgical interventions, wound size, ventilator days, and lengths of stay in the ICU and hospital. Hospital-acquired infections encompass pneumonia, bacteremia, urinary tract infections, and clostridium difficile infections not present upon admission, identified through culture results, treatment history, and consultation notes from infectious disease specialists.<br>● Patients in the early enteral nutrition group also had fewer ventilator days mean 5 vs 12<br>● There was no significance difference in mortality (13.2 vs 5.9%) for patients receiving early compared with late enteral feds

Author, year	Jiang 2020
Country	China
Study design	● Randomized, controlled, multicenter study
Configuration	● Research protocol was reviewed and approved by the Ethics Committee of Zhejiang Provincial People's Hospital<br>● Written informed consent was retrieved from all participators before inclusion

	• Registered in the Chinese Clinical Trial Registry
Population	• Inclusion criteria<br>○ Age >18 years<br>○ Acute Physiology and Chronic Health Evaluation II (APACHE II) score >8<br>○ Requirement to stay for at least 24 hours in the ICU.<br>○ Patients with sepsis as defined by the sepsis-3 diagnostic criteria, which include infection-causing life-threatening organ dysfunction and a Sequential Organ Failure Assessment (SOFA) score of ≥2.<br>• Exclusion criteria<br>○ Inability to test for AGI (reason unspecified).<br>○ Advanced cancer.<br>○ Any terminal stage disease.<br>• Characteristics<br>○ Mean age (70)<br>○ Male (69%)<br>○ Patient source<br>■ Internal medicine (79%)<br>○ BMI (20kg/m2)<br>○ Vasopressor (49.7%)<br>○ Albumin (29.3mmol/l)<br>○ Glucose (9.1mmol/l)<br>○ Serum lactate (3.3mmol/l)<br>○ Respiratory failure (75.4%)<br>○ AKI (20%)<br>○ Glucorticoid (20%)<br>○ SOFA score (9.8)<br>○ APACHE II score (20)
Intervention	• Early enteral nutrition EN (within 24 hours) administered based on stable hemodynamics. Infusion rate and daily intake depend on GRV and 20 kcal/kg/day target. For malnourished patients, SPN from day four if EN insufficient.
Control	• Control group = enteral nutrition after 24 hours

Outcomes	● SOFA scores within 24 hours of ICU admission.<br>● 28 day all cause mortality<br>○ 35.6%<br>● 60 day all cause mortality<br>○ 45.2%<br>● Patients with early enteral nutrition had a lower 28 and 60 day mortality rates than those in the control group<br>● Incidence of new infections (10.4%)

Author, year	Kaur 2005
Country	India
Study design	● Prospective cohort single center study
Configuration	● Was carried out in the Department of Surgery at the University College of Medical Sciences and the Guru Teg Bahadur Hospital, Delhi, from April 2000 to March 2002, after approval from the local ethical committee
Population	**Inclusion Criteria:**<br>● Subjects had to be aged between 20 and 70 years.<br>● Patients must have undergone emergency exploratory laparotomy.<br>● Subjects underwent laparotomy for nontraumatic perforation peritonitis.<br>● Patients were required to be malnourished at the time of presentation.<br>● Patients were required to give informed consent for inclusion in the study.<br>**Exclusion Criteria:**<br>● Patients with dementia were excluded.<br>● Individuals with diabetes were excluded.<br>● Patients with renal failure were excluded.<br>● Individuals with hepatic failure were excluded.<br>● Patients were considered malnourished if they met specific nutritional assessment criteria, and those who did not meet these criteria were likely excluded:<br>● Nutritional Risk Index (NRI) < 100.<br>● Current weight < 95% of the ideal weight and serum albumin at 39.2g/L.

	• Characterization<br>○ Age<br>■ 36<br>○ Males<br>■ 79%<br>○ Preoperative weight<br>■ 37.6<br>■ 46.60-49.39 kg
Intervention	• **Nasoenteric Tube Insertion:** At the end of surgery, a nasoenteric tube was inserted through the nasal route and advanced into the duodenum or jejunum.<br>• **Ryles Tube Placement:** A Ryles tube was placed in the stomach for decompression.<br>• **Postoperative Nutrition:** Patients in the test group (TG) received hospital kitchen-prepared feed through the nasojejunal tube 24 hours after surgery. The feed composition included 375 ml milk, 12.5 g sugar, 12.5 g butter, 12.5 g starch, 125 ml rice water, and half an egg, providing 500 kcal energy, 16.66 g protein, 43.5 g carbohydrate, and 30 g fat.<br>• **Feeding Rate:** The feed was initiated at a rate of 50 ml/h in the first 6 hours and gradually increased to 100 ml/h by the third postoperative day.<br>• **Nutritional Goals:** The nutritional goal was to deliver 35–45 kcal/kg/day and 1.5–2.0 g protein/kg/day.<br>• **Monitoring and Adjustments:** Feeding was discontinued for 6 hours for patients who developed abdominal distention, diarrhea, or vomiting, and then restarted at a slow rate. The nasogastric tube was removed when gastric aspirate was minimal or nil, and when patients started taking 2 L of feed per day, usually by the fourth or fifth postoperative day.
Control	• The control group received standard postoperative care
Outcomes	• The study found that early postoperative enteral nutrition through nasoenteric tubes resulted in a significant reduction in major septic complications compared to the control group. However, there was no significant difference in mortality or length of hospital stay between the two groups. In terms of nutritional status, the group receiving early enteral nutrition had a significantly less mean loss in weight and showed an

	increase in serum albumin levels and handgrip strength compared to the control group

Author, year	Lui 2018
Country	China
Study design	● Prospective cohort
Configuration	● Study approved by Nanjing Medical University's Ethics Committee<br>● Patients gave informed consent for peripheral venous blood collection<br>● Patients with sepsis on invasive mechanical ventilation were followed from January 2013 to March 2016, at the ICY of the Nanjing Hospital
Population	● Inclusion criteria<br>○ Age between 18 and 89 years<br>○ Requirement of norepinephrine within 48 hours of admission to the ICU<br>○ Diagnosis of "SIRS, sepsis, severe sepsis, or septic shock."<br>○ Received enteral nutrition (EN) supplementation during their ICU stay<br>● Exclusion criteria<br>○ Age less than 18 years or greater than 89 years.<br>○ Not requiring norepinephrine within 48 hours of admission.<br>○ Not being supplied with EN during their ICU stay.
Intervention	● Patients with sepsis who received enteral nutrition treatment within 48 hours of being admitted to the ICU were assigned to the EEN group
Control	● Those who received enteral nutrition treatment after 48 hours of admission to the ICU were assigned to the DEN group<br>●
Outcomes	● The 28 day mortality of the EEN group was 37.14%, and that of the DEN group was 42.86%. There was no significant difference between the 2 groups.<br>● Duration of mechanical ventilation

	○ EEN – 9.49 days<br>○ DEN – 11.61 days

Author, year	Patel 2016
Country	United states
Study design	● Retrospective study
Configuration	● Data was collected from April 1, 2011, through March 21, 2012<br>● The study was approved by the Medical College of Wisconsin institutional review board and met all national guidelines for protection of human studies
Population	● Inclusion criteria<br>○ Patients must be 18 years of age or older.<br>○ Patients must have been admitted to the medical intensive care unit (MICU) between the period of April 1, 2011, through March 31, 2012.<br>○ Patients must have a documented MICU admission diagnosis of septic shock that required vasopressor support.<br>○ Patients must have been placed on mechanical ventilation either upon admission to the MICU or within 24 hours of admission, and they must have remained on mechanical ventilation for more than 48 hours.<br>● Exclusion criteria<br>○ Documented Contraindications for Enteral Feeding: Patients who had a documented contraindication for enteral feeding were excluded from the study. Contraindications for enteral feeding include:<br>○ Bowel obstruction.<br>○ Protracted ileus.<br>○ Intractable vomiting.<br>○ Major upper gastrointestinal bleed.<br>○ Bowel surgery within the previous 30 days.

	● Characteristics<br>○ Median age (35-75)<br>○ Male (59%)<br>○ Median BMI (22-46)<br>○ BMI (20+-5.8 kg/m2)<br>○ Vasopressor (49.7%)<br>○ Sepsis source<br>■ Pulmonary (51.6%)<br>■ Urogenital (12.6%)<br>○ Vasopressors<br>■ Norepinephrine 71%<br>■ Epinephrine 4%<br>■ Multiple 25%<br>○ Serum lactate (1.5-8.8 mmol/l)<br>○ Median Kcal/d while on vasopressors (0-907)
Intervention	● **Trophic Feeding**: Patients within the intervention group who were receiving trophic feeding, which is defined as minimal enteral nutrition typically provided at a rate of 10 to 30 mL/h and considered to be less than 600 kcal/d (but more than 1 kcal/d) in this study.<br>● **Full EN (Enteral Nutrition)**: Patients within the intervention group who were receiving full enteral nutrition, which would provide them with their complete caloric and nutritional requirements based on their clinical condition.
Control	● Patients without enteral nutrition within 48 hours of MICU admission. Study compares outcomes of enteral nutrition (trophic/full EN) recipients to non-recipients, using control as a reference.
Outcomes	● The study examined in-hospital mortality, duration of mechanical ventilation (DOMV), length of stay (LOS), and feeding intolerance, which encompasses clinical issues like diarrhea, vomiting, gastrointestinal bleeding, ileus, and high gastric residual volume. Complications include nonocclusive mesenteric ischemia and bowel necrosis. Aspiration pneumonia was identified by clinical and radiographic signs with tube feeding in airway suctioning.<br>● Mortality

	○ Intervention 21%<br>○ Control 33.3%<br><br>● Days of mechanical ventilation<br>○ Intervention (2-15)<br>○ Control (5-27)<br><br>● Ileus<br>○ Intervention 91.5%<br>○ Control 86.6%

Author, year	Malhotra 2004
Country	India
Study design	● Randomized controlled single center study
Configuration	● Was carried out in a surgical unit in a medical college hospital between May 2000 and February 2003. The study proposal was studied and approved by the department review committee.
Population	**Inclusion Criteria:**<br><br>● Patients with enteric perforations were included in the study.<br>● Patients who underwent emergency surgery after relevant investigations.<br>● Patients who received routine administration of intravenous fluids and anti-microbial agents prior to surgery.<br>● Patients who underwent routine naso-gastric tube aspiration.<br>● Patients who had not undergone ileostomy after surgery.<br>● Inclusion in the study required obtaining informed consent from the eligible patients.<br>● The study proposal was studied and approved by the department review committee.<br><br>**Exclusion Criteria:**<br><br>● Patients who underwent ileostomy following surgery were excluded from the study.

	● Patients who did not provide informed consent were excluded
Intervention	● Naso-gastric tube used for both feeding and aspiration.<br>● Subjects in Group A received 100 grams of a balanced diet formula dissolved in 500 ml of gram dry weight (GDW) 5% (600 Calories) slowly at the rate of 50 ml/hour via an intravenous drip set connected to a naso-gastric tube.<br>● The rate of feeding was adjusted or feeding was stopped if the patient developed intolerable distension, uneasiness, vomiting, hiccough, or abdominal pain.<br>● Patients received another 300-400 calories in the form of intravenous dextrose.<br>● From the fifth postoperative day, in addition to enteral feeds, patients were transitioned to complete oral feeds in the form of semi-solid diet.<br>● Between the eighth and tenth day, the naso-gastric tube was removed, and patients were kept on intravenous patency line.<br>● Patients were closely monitored for signs of a leak from the repaired perforation of the gut.
Control	● Patients in Group B were assessed for the feasibility of oral intake on the fifth postoperative day.<br>● Those found suitable were given sips of an appetizing liquid, and those tolerating the sips graduated to 500-ml liquids and then semi-solids over the next two days.<br>● Patients who did not tolerate oral feed stayed on intravenous fluids until they could take feeds orally.
Outcomes	● **Risk of Complications:**<br>● Group A demonstrated a lower risk of complications such as leak, wound dehiscence, wound infection, septicaemia, pneumonia, and death compared to Group B.

- However, these differences were not statistically significant

- **Relative Risk and Odds Ratios:**
  - The relative risk and odds ratios for major complications were lower for Group A.
  - Again, these differences were not statistically significant.
- **Risk of Minor Complications:**
  - The risk of minor complications such as vomiting, diarrhea, and abdominal distension was higher in Group A, although not statistically significant.
- **Duration of Major Complications:**
  - The duration in which major complications were controlled was significantly lower in patients receiving early enteral nutrition (Group A).
  - This was reflected in the fewer number of 'man-days' lost.
- **Nutritional Parameters:**
  - Patients in the study group (Group A) showed better general condition, less weight loss, better nitrogen balance, and higher serum albumin compared to those who received conventional management (Group B)
  - Alimentation, especially through enteral feeding, played a significant role in maintaining better nutritional status
- **Duration of Hospital Stay:**
  - Although the mean duration of stay, both in general and in the ICU, was lower in the study group, these differences were not statistically significant

	● **Control of Complications in Enterally Fed Patients:**<br>● Although the incidence of major complications was reduced in Group A, the differences were not statistically significant.<br>● However, the reduction in 'Man-Days' of major complications was substantial, suggesting that complications in enterally fed patients were controlled more quickly than in conventionally managed patients<br>● The study emphasizes that the incidence of complications cannot be the sole parameter for the usefulness of enteral feeding in emergency surgery for gut perforations, given the inherent complications in conditions like faecal peritonitis<br>● The study strongly recommends early enteral nutrition in operated cases of gut perforations.

Author, year	Sun 2019
Country	China
Study design	● Prospective, single center and randomized clinical trial
Configuration	● Randomization utilized computer generated random numbers followed by the remainder grouping method (divided by two) to assign patients to receive early enteral nutrition (EEN) or delayed enteral nutrition (DEN) groups. Treatment allocation remained concealed until patient inclusion<br>● Single blinded<br>● The protocol was approved by the Institutional Ethics Committee of Nanjing First Hospital, informed consent was obtained from each patient's first degree

	relatives.<br>● This study was registered at clinicaltrials.gov
Population	● Inclusion criteria<br>○ Patients aged 18-70 years<br>○ Admitted into the ICU of Nanjing First Hospital<br>○ Diagnosis of sepsis according to the surviving sepsis guidelines<br>● Exclusion criteria<br>○ Patients with ileus or digestive tract hemorrhage<br>○ Patients with inflammatory bowel disease<br>○ Patients with severe abdominal hypertension<br>○ Patients with chronic organ dysfunction<br>○ Patients with malnutrition<br>○ Patients with immunodeficiency<br>○ Patients with a history of long term use of hormones<br>● Characteristics<br>○ Mean age 58 years<br>○ Males 68%<br>○ Mean BMI 25<br>○ SOFA 9.25<br>○ APACHE II 22<br>○ Abdominal infection 47%<br>○ Thoracic/pulmonary infection 36%
Intervention	● Early enteral nutrition (EEN) Group: in this group, enteral feeding was initiated within the first 24-48 hours after admission<br>● 26 recieved ENN<br>● The enteral nutrition involved the use of specific feeding tubes, formula types (peptide-based and whole protein formula), defined goals for caloric and protein intake, feeding rates, and adjustments based on patient tolerance. Additionally, the text mentions that parenteral nutrition (PN) was supplemented if enteral feedings alone could not meet a certain percentage of energy and protein requirements after 7 days, with specific caloric intake and calorie/nitrogen ratio defined for PN.
Control	● Delayed Enteral Nutrition (DEN) Group: In this group, enteral feeding was initiated on the 4th day after admission.<br>● 27 received DEN

Outcomes	● Sequential organ failure assessment (SOFA)<br>○ Day 7: 4.4<br>● 28 days mortality<br>● Days in the ICU<br>● Days of mechanical ventilation<br>○ 5.8 days<br>● Number of patients receiving continuous renal replacement therapy were also recorded (CRRT)<br>○ 13 %<br>● Over 28 days of admission, 15.4% of patients in the EEN group (4 out of 26) and 22.2% of patients in the DEN group (6 out of 27) succumbed to MODS or infectious complications.

Author, year	Márquez 2023
Country	• México
Study design	• Prospective cohort study
Population	● Inclusion criteria:<br>○ Septic shock (defined as Sepsis-3: mean arterial pressure less than 65 mm Hg, lactate levels < 2 mmol/L, sustained vasopressor therapy requirement).<br>○ Need for mechanical ventilation independently.<br>● Exclusion criteria:<br>○ Initiation of mechanical ventilation more than 48 hours after ICU admission, patients diagnosed with diseases having a baseline survival of less than 6 months.<br>○ Patients from the COVID-19 respiratory therapy unit.
Intervention	● Early enteral nutrition (within 48 hours)<br>○ 110
Control	● Delayed enteral nutrition<br>○ 21
Outcomes	● Days of mechanical ventilation: early nutrition 2 (0-5), late nutrition 5 (2-9), p=0.012.<br>● Mortality: early nutrition 18 (13;74), late nutrition 6 (4;58), p=0.21.

Author, year	Slone 2016
Country	United States
Study design	• Retrospective cohort
Configuration	• Patients categorized into early (EN < 48 hours) and late (EN > 48 hours) groups for analysis: 469 early, 211 late.<br>• Data were collected from January 1, 2005, to July 31, 2015
Population	• Inclusion criteria<br>○ Age >18 years.<br>○ Acute Physiology and Chronic Health Evaluation II (APACHE II) score >8.<br>○ Requirement to stay for at least 24 hours in the ICU.<br>○ Patients with sepsis as defined by the sepsis-3 diagnostic criteria, which include infection-causing life-threatening organ dysfunction and a Sequential Organ Failure Assessment (SOFA) score of ≥2.<br>• Exclusion criteria<br>○ Inability to test for AGI (reason unspecified).<br>○ Advanced cancer.<br>○ Any terminal stage disease.
Intervention	• Early enteral nutrition EN (within 24 hours) administered based on stable hemodynamics. Infusion rate and daily intake depend on GRV and 20 kcal/kg/day target. For malnourished patients, SPN from day four if EN insufficient.
Control	• Control group = enteral nutrition after 24 hours
Outcomes	• Hospital mortality didn't differ significantly between early and late EN groups, considering age, sex, CCI, and SOFA score.<br>• Age and SOFA score showed significant associations with hospital mortality (p<.001). Younger individuals had lower mortality, and higher SOFA scores increased mortality risk.

	● Sex and CCI didn't impact hospital mortality.

Author, year	Wang 2023
Country	China
Study design	● Retrospective cohort study
Configuration	● ICU admissions data from Beth Israel Deaconess Medical Center (2001-2012) using MIMIC-III, approved by ethical standards.
Population	● Inclusion criteria<br>○ Patients over 18 years old.<br>○ Patients with a diagnosis of sepsis.<br>○ Patients receiving EN (Enteral Nutrition) during the ICU stay.<br>○ Sepsis diagnosis based on the Angus methodology, which includes documented infection and acute organ dysfunction according to the International Classification of Diseases, Ninth Revision, Clinical Modification.<br>● Exclusion criteria<br>○ Patients who stayed in the ICU less than 24 hours<br>● Characterization<br>○ Age mean 66 years<br>○ BMI (23-33 kg/m2)<br>○ Service unit<br>■ CCU7.75%<br>■ MICU 51.5%<br>■ TSICU 15.1%<br>○ Admission<br>■ Elective 4.4%<br>■ Urgent 1.4%<br>○ Lactate (1.3-3.5)<br>○ SOFA 11<br>○ Comorbidities<br>■ CHF 25.3%

	■ CA 33.4%<br>■ Hypertension 17.98%<br>■ Paralysis 8.8%<br>■ COPD 26%<br>■ Diabetes 31%<br>■ Malignancy 8.7%<br>■ CKD 21.5%<br>■ Vasopressor use in the first 24 hours.<br>● 43%
Intervention	● Early enteral nutrition<br>○ Within 53 hours of admission at the ICU
Control	● Delayed enteral nutrition<br>○ After 53 hours of admission at the ICU
Outcomes	● The primary outcome was the incidence of AKI<br>○ After propensity score matching, the incidence of AKI for the EEN group was significantly lower (74.2% vs 12.1%, P >0.001)<br>● Secondary outcomes included AKI stages; 28-day-mortality, number of ventilator renal replacement therapy<br>○ Compared with DEN patients larger percentage of the EEN patients has stage 1 AKI (15% vs 12.1%, p<0.001), and a smaller percentage of the EEN patients had stage 3 AKI (29% vs 42.4 %)<br>● The duration of mechanical ventilation (ventilation-free days on day 28 of 23.87 days vs. 21.33 days, p<0.001)<br>● There was no difference in 28-day-mortality

Author, year	Sun 2017
Country	China

<table>
<tr><td>Study design</td><td>

- Retrospective review

</td></tr>
<tr><td>Configuration</td><td>

- The study received approval from the institutional review board of the Nanjing First Hospital, and written informed consent was not required because it was a retrospective study.

</td></tr>
<tr><td>Population</td><td>

- Inclusion criteria
  - Patients included in the study must be 18 years of age or older.
  - Patients must have been admitted to the surgical Intensive Care Unit (ICU) of Nanjing First Hospital.
  - Patients should have a diagnosis of sepsis that aligns with the surviving sepsis guidelines.
  - Patients must have an ICU stay of at least 48 hours.
- Exclusion criteria
  - Chronic Organ Dysfunction
  - Coagulation Dysfunction
  - Diabetes Mellitus
  - Malnutrition
  - Immunodeficiency
  - Patients who had received artificial nutrition (enteral or parenteral nutrition) before their admission to the ICU were not included in the study.
- Characterization
  - Age mean 72
  - Male 58%
  - Etiology
    - Abdominal infection 65%
    - Thoracic/pulmonary infection 26%
  - BMI (21-25.5)
  - SOFA (6-12.5)

</td></tr>
<tr><td>Intervention</td><td>

- **Early Enteral Nutrition (EEN) Group**: In this group, enteral nutrition was initiated during the first 48 to 72 hours after admission to the hospital. Patients in this group received either a peptide-based formula (Peptisorb) in the first 24 to 48 hours, followed by a whole protein formula (Nutrison Fibre) if they were tolerant. The goal intake for energy was set at 20 to 25 kcal/kg/d, and the protein intake was calculated as 1.5 g/kg/d. The feeding rate was initiated at 15 to 20 mL/h and gradually increased by 15

</td></tr>
</table>

	to 20 mL every 6 to 8 hours using a pump<br>● 0-70% energy from glucose, lipid supply matched triglycerides. IV solution included vitamins, electrolytes, insulin, and trace elements.<br>● 46 (43%) patients received EEN
Control	● **Delayed Enteral Nutrition (DEN) Group**: Patients in this group received enteral nutrition on the 4th day or later after admission, as opposed to the earlier initiation in the EEN group. The text does not provide specific details about the type of formula used for delayed enteral nutrition.<br>● 36 (33.6%) received DEN
Outcomes	● 28 day mortality, duration of ICU stay, and the incidence of MODS were also recorded<br>● At 28 days of admission, 5 of 46 patients (10.9%) in EEN group, 8 of 36 patients (22.2%)in DEN group<br>● SOFA<br>○ Day 3<br>■ EEN 8<br>■ DEN 8<br>○ Day 7<br>■ EEN 7<br>■ DEN 7<br>○ Day 14<br>■ EEN 5<br>■ DEN 5.5

Author, year	Ortiz-Reyes 2022
Country	Argentina, Australia, Brazil, Canada, Greece, Hong Kong, and more
Study design	• Prospective cohort study
Configuration	• Study protocol approved by Queen's University Ethics Board, local institutional review boards (IRBs) for RCT approval, and informed consent, with waivers as needed.<br>• Data collected from 2018 to 2021 in 100 ICUs across various countries including Argentina, Australia, Brazil, Canada, Greece, Hong Kong, and more.
Population	• Inclusion criteria<br>○ Age: Patients should be at least 18 years old.<br>■ Nutritional Risk: Patients should be considered nutritionally high-risk, which can be defined by meeting at least one of the following criteria:<br>■ (2a) Body Mass Index (BMI) less than 25 or greater than 35.<br>■ (2b) Moderate or severe malnutrition (as defined by local assessments).<br>■ (2c) Clinical frailty scale score of 5 or higher, indicating frailty.<br>■ (2d) SARC-F score of 4 or higher, indicating

        - sarcopenia.
        - (2e) Expected mechanical ventilation (MV) for more than 96 hours.
    - Mechanical Ventilation: Patients must require mechanical ventilation with an actual or expected total duration of more than 48 hours.
    - Eligibility for Nutrition Support: Patients must be eligible to receive nutrition support, which can include enteral nutrition (EN), parenteral nutrition (PN), a combination of EN and PN, or amino acids only.
- Exclusion criteria
    - Pregnancy.
    - More than 96 continuous hours of mechanical ventilation before enrollment.
    - Expected death or withdrawal of life-sustaining treatments within 7 days from enrollment.
    - The responsible clinician believes the patient requires either usual or high protein intake (lack of equipoise).
    - The patient requires PN only, and the site does not have products to achieve the high protein dose.
- Characteristics
    - Age mean 57
    - Gender Male 56%
    - BMI 28.2
    - Type of admission
        - Medical 87%
    - Patients with septic shock 55.1%
    - SOFA scores mean 9.4
    - Frailty 89%
    - Sarcopenia 100%

Intervention	• Early enteral nutrition EEN (<48 h of ICU admission)<br>• 526 received EEN<br>• In the study, 91% of patients received enteral nutrition (EN), predominantly through gastric feeding tubes. Various feeding practices were identified, including low-rate initiation in 47% and trophic feeds in 2%. Most used polymeric EN (81%), and indirect calorimetry was employed in 6.9% of cases. EN was typically initiated within a median of 16 hours after vasopressor initiation.
Control	• Delayed enteral nutrition (>48 h of ICU admission)<br>• 100 received DEN<br>• Dietitian-led EN initiation was observed at a median of 59.4 hours (IQR 43.2–73.6) compared to a range of 2.3–234.6 hours in other cases, demonstrating a statistically significant difference
Outcomes	• Enteral feeding intolerance (EFI) events, as defined locally by participating ICUs, encompassed issues like high gastric residuals, abdominal distention, vomiting, diarrhea, and discomfort. EFI events were recorded post-EN initiation until discontinuation.<br>• In mechanically ventilated patients with circulatory shock, the study aimed to assess the impact of early enteral nutrition (EEN) versus delayed enteral nutrition (DEN) on the primary outcome of PODS+death at 28 days. PODS+death is the presence of organ dysfunction along with receiving vasopressors, dialysis, and/or mechanical ventilation, coupled with patient mortality at specific assessment time points. Secondary outcomes included various measures such as ICU length of stay, hospital length of stay, mechanical ventilation duration, readmission rate, vasopressor use duration, renal replacement therapy, ICU and 60-day mortality, and enteral feeding intolerance (EFI) rates. Additional measures included the count of ICU-free days and vasopressor-free days out of 28 since ICU admission.

Author, year	Koga 2018
Country	Japan
Study design	• Retrospective cohort
Configuration	• Conducted Institutional Review Board-approved retrospective analysis of sepsis cases in Yamaguchi ICU, reviewing adult patient records from January 2010 to August 2017.
Population	• Inclusion criteria<br>○ Subjects meeting Sepsis-3 definition<br>• Exclusion criteria<br>○ < 18 years<br>○ Pregnant females<br>○ Those without abdominal CT within 24 hours<br>○ Individuals with iliopsoas abscess<br>○ Hematoma.<br>○ Excluded patients who died within 48 hours due to low probability of EEN receipt.<br>• Characteristics<br>○ Mean age (71)<br>○ Male (66%)<br>○ BMI (18.1-24.2 kg/m2)<br>○ SOFA score<br>■ Day 1 (9.5)<br>○ Septic shock (51%)<br>○ Infection source<br>■ Respiratory (35%)<br>■ Abdominal (23%)
Intervention	• individually prescribed enteral nutrition (EN) by attending physicians, typically initiated after hemodynamic stabilization with or without vasopressor use, starting at a low flow rate via a gastric tube and gradually increasing as necessary.<br>• Supplemental protein in addition to standard enteral and parenteral

	formulas was not applied
Control	• "delayed enteral nutrition (DEN) group." These patients did not receive early enteral nutrition (EEN) as the intervention group did.
Outcomes	• SOFA score day 1<br>○ Non sarcopenia<br>■ EEN 9<br>■ DEN 10<br>○ Sarcopenia<br>■ EEN 7<br>■ DEN 9<br>• In-hospital mortality<br>○ Non sarcopenia<br>■ EEN 16%<br>■ DEN 16%<br>○ Sarcopenia<br>■ EEN 9%<br>■ DEN 34%

Study	Reason for exclusion
**Artinian, 2006**[1]	Excluded because engaged a different population.
**Altintas, 2011**[2]	Excluded because administered a non-relevant comparison.
**Casaer,2011**[3]	Excluded because administered a non-relevant intervention.
**Cha, 2022**[4]	Excluded because administered a non-relevant intervention.
**Elke, 2008**[5]	Excluded because administered a non-relevant comparison.
**Elke, 2013**[6]	Excluded because administered a non-relevant intervention.
**El Meligy, 2023**[7]	Excluded because engaged a different population.
**Ibrahim, 2002**[8]	Excluded because administered a non-relevant comparison.
**Khalid, 2010**[9]	Excluded because engaged a different population.
**Kompan, 2004**[10]	Excluded because engaged a different population.
**Mancl, 2013**[11]	Excluded because administered a non-relevant comparison.
**Nguyen,2008**[12]	Excluded because engaged a different population.
**Pardo, 2023**[13]	Excluded because engaged a different population.
**Radrizzani, 2006**[14]	Excluded because administered a non-relevant comparison.
**Rai, 2010**[15]	Excluded because administered a non-relevant comparison.

Reignier, 2018[16]	Excluded because administered a non-relevant comparison.
Reignier, 2023[17]	Excluded because administered a non-relevant comparison.
Sim, 2021[18]	Excluded because engaged a different population.
Singh, 1998[19]	Excluded because engaged a different population.
Shan, 2021[20]	Excluded because administered a non-relevant intervention.
Yan, 2023[21]	Excluded because administered a non-relevant comparison.
Yuan Y, 2011[21]	Excluded because administered a non-relevant intervention.

REFERENCES EXCLUDED STUDIES.

[1] Artinian V, Krayem H, DiGiovine B. Effects of Early Enteral Feeding on the Outcome of Critically Ill Mechanically Ventilated Medical Patients. Chest 2006;129:960–7. https://doi.org/10.1378/chest.129.4.960.

[2] Altintas ND, Aydin K, Türkoğlu MA, Abbasoğlu O, Topeli A. Effect of Enteral Versus Parenteral Nutrition on Outcome of Medical Patients Requiring Mechanical Ventilation. Nutr Clin Pract 2011;26:322–9. https://doi.org/10.1177/0884533611405790.

[3] Casaer MP, Mesotten D, Hermans G, Wouters PJ, Schetz M, Meyfroidt G, et al. Early versus Late Parenteral Nutrition in Critically Ill Adults. N Engl J Med 2011;365:506–17. https://doi.org/10.1056/NEJMoa1102662.

[4] Cha J-K, Kim H-S, Kim E-J, Lee E-S, Lee J-H, Song I-A. Effect of Early Nutritional Support on Clinical Outcomes of Critically Ill Patients with Sepsis and Septic Shock: A Single-Center Retrospective Study. Nutrients 2022;14:2318. https://doi.org/10.3390/nu14112318.

[5] Elke G, Schädler D, Engel C, Bogatsch H, Frerichs I, Ragaller M, et al. Current practice in nutritional support and its association with mortality in septic patients—Results from a national, prospective, multicenter study*. Crit Care Med 2008;36:1762–7. https://doi.org/10.1097/CCM.0b013e318174dcf0.

[6] Elke G, Kuhnt E, Ragaller M, Schädler D, Frerichs I, Brunkhorst FM, et al. Enteral nutrition is associated with improved outcome in patients with severe sepsis. Medizinische Klin - Intensivmed Und Notfallmedizin 2013;108:223–33. https://doi.org/10.1007/s00063-013-0224-4.

[7] El Meligy BS, El-sherbini SA, Soliman MM, abd El-Ghany HM, Ahmed ES. Early enteral versus early parenteral nutrition in critically ill patients with respiratory distress: a case–control study. Egypt Pediatr Assoc Gaz 2023;71:20. https://doi.org/10.1186/s43054-023-00162-1.

[8] Ibrahim E, Mehringer L, Prentice D, Sherman G, Schaiff R, Fraser V, et al. Early versus late enteral feeding of mechanically ventilated patients: results of a clinical trial. J Parenter Enter Nutr 2002;26:174–81. https://doi.org/10.1177/0148607102026003174.

[9] Khalid I, Doshi P, DiGiovine B. Early Enteral Nutrition and Outcomes of Critically Ill Patients Treated With Vasopressors and Mechanical Ventilation. Am J Crit Care 2010;19:261–8. https://doi.org/10.4037/ajcc2010197.

[10] Kompan L, Vidmar G, Spindler-Vesel A, Pečar J. Is early enteral nutrition a risk factor for gastric intolerance and pneumonia? Clin Nutr 2004;23:527–32. https://doi.org/10.1016/j.clnu.2003.09.013.

[11] Mancl EE, Muzevich KM. Tolerability and Safety of Enteral Nutrition in Critically Ill Patients Receiving Intravenous Vasopressor Therapy. J Parenter Enter Nutr 2013;37:641–51. https://doi.org/10.1177/0148607112470460.

[12] Nguyen NQ, Fraser RJ, Bryant LK, Burgstad C, Chapman MJ, Bellon M, et al. The impact of delaying enteral feeding on gastric emptying, plasma cholecystokinin, and peptide YY concentrations in critically ill patients*. Crit Care Med 2008;36:1469–74.

https://doi.org/10.1097/CCM.0b013e31816fc457.

[13] Pardo E, Lescot T, Preiser J-C, Massanet P, Pons A, Jaber S, et al. Association between early nutrition support and 28-day mortality in critically ill patients: the FRANS prospective nutrition cohort study. Crit Care 2023;27:7. https://doi.org/10.1186/s13054-022-04298-1.

[14] Radrizzani D, Bertolini G, Facchini R, Simini B, Bruzzone P, Zanforlin G, et al. Early enteral immunonutrition vs. parenteral nutrition in critically ill patients without severe sepsis: a randomized clinical trial. Intensive Care Med 2006;32:1191–8. https://doi.org/10.1007/s00134-006-0238-y.

[15] Rai SS, O'Connor SN, Lange K, Rivett J, Chapman MJ. Enteral nutrition for patients in septic shock: a retrospective cohort study. Crit Care Resusc 2010;12:177–81. https://doi.org/10.1016/S1441-2772(23)01501-6.

[16] Reignier J, Boisramé-Helms J, Brisard L, Lascarrou J-B, Ait Hssain A, Anguel N, et al. Enteral versus parenteral early nutrition in ventilated adults with shock: a randomised, controlled, multicentre, open-label, parallel-group study (NUTRIREA-2). Lancet 2018;391:133–43. https://doi.org/10.1016/S0140-6736(17)32146-3.

[17] Reignier J, Plantefeve G, Mira J-P, Argaud L, Asfar P, Aissaoui N, et al. Low versus standard calorie and protein feeding in ventilated adults with shock: a randomised, controlled, multicentre, open-label, parallel-group trial (NUTRIREA-3). Lancet Respir Med 2023;11:602–12. https://doi.org/10.1016/S2213-2600(23)00092-9.

[18] Sim J, Hong J, Na EM, Doo S, Jung YT. Early supplemental parenteral nutrition is associated with reduced mortality in critically ill surgical patients with high nutritional risk. Clin Nutr 2021;40:5678–83. https://doi.org/10.1016/j.clnu.2021.10.008.

[19] Singh G, Ram RP, Khanna SK. Early postoperative enteral feeding in patients with nontraumatic intestinal perforation and peritonitis. J Am Coll Surg 1998;187:142–6. https://doi.org/10.1016/S1072-7515(98)00154-9.

[20] Shah FA, Kitsios GD, Yende S, Dunlap DG, Scholl D, Chuan B, et al. A Pilot Double-Blind Placebo-Controlled Randomized Clinical Trial to Investigate the Effects of Early Enteral Nutrients in Sepsis. Crit Care Explor 2021;3:e550. https://doi.org/10.1097/CCE.0000000000000550.

[21] Yuan Y, Ren J, Gu G, Chen J, Li J. Early Enteral Nutrition Improves Outcomes of Open Abdomen in Gastrointestinal Fistula Patients Complicated With Severe Sepsis. Nutr Clin Pract 2011;26:688–94. https://doi.org/10.1177/0884533611426148.

Table S3. **Summary of findings table for RCTs.**

Early Enteral Nutrition (Within 48 Hours) For Patients with Sepsis or Septic Shock

Patient or population: Patients over 16 years of age, who had been admitted to intensive care unit (ICU) for at least 48 hours for sepsis or septic shock regardless etiology.
Setting: Intensive care unit (ICU)
Intervention: Early enteral nutrition (within 48 hours)
Comparison: No nutrition or delayed enteral nutrition (after 48 hours).

Outcomes	Anticipated absolute effects* (95% CI)		Relative effect (95% CI)	№ of participants (studies)	Certainty of the evidence (GRADE)
	Risk with no nutrition or delayed enteral nutrition	Risk with early enteral nutrition			
Mortality at 28 days	291 per 1.000	**303 per 1.000** (156 to 502)	**OR 1.06** (0.45 to 2.46)	108 (2 RCTs)	⨁⨁◯◯ Low[a]
In-hospital Mortality assessed with: Mortality in ICU or Prior to Discharge	173 per 1.000	**126 per 1.000** (75 to 204)	**OR 0.69** (0.39 to 1.23)	381 (4 RCTs)	⨁◯◯◯ Very low[b,c]
Days of mechanical ventilation	-- per 1.000	**-Infinity per 1.000** (-- to --)	**MD -2.65** (-4.44 to -0.86)	(1 RCT)	⨁⨁◯◯ Low[d]
Renal replacement therapy	338 per 1.000	**320 per 1.000** (173 to 509)	**OR 0.92** (0.41 to 2.03)	139 (3 RCTs)	⨁⨁◯◯ Low[e]
Ileus	0 per 1.000	**0 per 1.000** (0 to 0)	not estimable	31 (1 RCT)	⨁⨁◯◯ Low[f,g]
Diarrhea	164 per 1.000	**304 per 1.000** (184 to 460)	**OR 2.23** (1.15 to 4.34)	255 (2 RCTs)	⨁◯◯◯ Very low[b,h,i]
SOFA Score	-- per 1.000	**-Infinity per 1.000** (-- to --)	**MD -1.64** (-2.60 to -0.68)	(2 RCTs)	⨁⨁◯◯ Low[j]
Ventilator-associated pneumonia	63 per 1.000	**22 per 1.000** (1 to 371)	**OR 0.33** (0.01 to 8.83)	31 (1 RCT)	⨁⨁◯◯ Low[k]

Table S3. **Summary of findings table for RCTs.**

Early Enteral Nutrition (Within 48 Hours) For Patients with Sepsis or Septic Shock

Patient or population: Patients over 16 years of age, who had been admitted to intensive care unit (ICU) for at least 48 hours for sepsis or septic shock regardless etiology.
Setting: Intensive care unit (ICU)
Intervention: Early enteral nutrition (within 48 hours)
Comparison: No nutrition or delayed enteral nutrition (after 48 hours).

Outcomes	**Anticipated absolute effects*** (95% CI)		Relative effect (95% CI)	№ of participants (studies)	Certainty of the evidence (GRADE)
	**Risk with no nutrition or delayed enteral nutrition**	**Risk with early enteral nutrition**			

***The risk in the intervention group** (and its 95% confidence interval) is based on the assumed risk in the comparison group and the **relative effect** of the intervention (and its 95% CI).

CI: confidence interval; **OR:** odds ratio

GRADE Working Group grades of evidence
High certainty: we are very confident that the true effect lies close to that of the estimate of the effect.
Moderate certainty: we are moderately confident in the effect estimate: the true effect is likely to be close to the estimate of the effect, but there is a possibility that it is substantially different.
Low certainty: our confidence in the effect estimate is limited: the true effect may be substantially different from the estimate of the effect.
Very low certainty: we have very little confidence in the effect estimate: the true effect is likely to be substantially different from the estimate of effect.

Explanations

a. Downgraded by two levels for imprecision.
b. Downgraded by one level for serious limitations on blinding, some concerns on the randomization process, and selection of the reported result.
c. Downgraded by two levels for imprecision.
d. Downgraded by two levels for imprecision.
e. Downgraded by two levels for imprecision.
f. Downgraded by on level for serious limitations on blinding, some concerns due to randomization process.
g. Downgraded by one level for not optimal information size.
h. Downgraded by one level for serious limitations on substantial heterogeneity (I2> 40%).
i. Downgraded by two levels for imprecision.
j. Downgraded by two levels for imprecision.
k. Downgraded by two levels for imprecision.

Table S4. Summary of findings table for NRS

Early Enteral Nutrition (Within 48 Hours) For Patients with Sepsis or Septic Shock

Patient or population: Patients over 16 years of age, who had been admitted to intensive care unit (ICU) for at least 48 hours for sepsis or septic shock regardless etiology.
Setting: Intensive care unit (ICU)
Intervention: Early enteral nutrition (within 48 hours)
Comparison: No nutrition or delayed enteral nutrition (after 48 hours).

Outcomes	**Anticipated absolute effects*** (95% CI)		Relative effect (95% CI)	№ of participants (studies)	Certainty of the evidence (GRADE)
	**Risk with no nutrition or delayed enteral nutrition**	**Risk with early enteral nutrition**			
Mortality at 28 days	271 per 1.000	**248 per 1.000** (216 to 286)	**OR 0.89** (0.74 to 1.08)	2570 (5 observational studies)	⊕⊕◯◯ Low[a]
In-hospital Mortality assessed with: Mortality in ICU or Prior to Discharge	281 per 1.000	**258 per 1.000** (212 to 306)	**OR 0.89** (0.69 to 1.13)	1779 (7 observational studies)	⊕◯◯◯ Very low[b,c]
Days of mechanical ventilation	-- per 1.000	**-Infinity per 1.000** (-- to --)	**DM -2.94** (-3.64 to -2.23)	(6 observational studies)	⊕⊕◯◯ Low[d]
Renal replacement therapy	23 per 1.000	**21 per 1.000** (11 to 40)	**OR 0.89** (0.46 to 1.73)	1636 (1 observational study)	⊕◯◯◯ Very low[e]
Ileus	67 per 1.000	**39 per 1.000** (4 to 265)	**OR 0.57** (0.06 to 5.04)	66 (2 observational studies)	⊕◯◯◯ Very low[f,g]
SOFA score	-- per 1.000	**-Infinity per 1.000** (-- to --)	**MD -1.08** (-1.90 to -0.26)	(2 observational studies)	⊕◯◯◯ Very low[a,h]

***The risk in the intervention group** (and its 95% confidence interval) is based on the assumed risk in the comparison group and the **relative effect** of the intervention (and its 95% CI).

CI: confidence interval; **OR:** odds ratio

Table S4. Summary of findings table for NRS

Early Enteral Nutrition (Within 48 Hours) For Patients with Sepsis or Septic Shock

Patient or population: Patients over 16 years of age, who had been admitted to intensive care unit (ICU) for at least 48 hours for sepsis or septic shock regardless etiology.
Setting: Intensive care unit (ICU)
Intervention: Early enteral nutrition (within 48 hours)
Comparison: No nutrition or delayed enteral nutrition (after 48 hours).

Outcomes	Anticipated absolute effects* (95% CI)		Relative effect (95% CI)	№ of participants (studies)	Certainty of the evidence (GRADE)
	Risk with no nutrition or delayed enteral nutrition	Risk with early enteral nutrition			

GRADE Working Group grades of evidence
High certainty: we are very confident that the true effect lies close to that of the estimate of the effect.
Moderate certainty: we are moderately confident in the effect estimate: the true effect is likely to be close to the estimate of the effect, but there is a possibility that it is substantially different.
Low certainty: our confidence in the effect estimate is limited: the true effect may be substantially different from the estimate of the effect.
Very low certainty: we have very little confidence in the effect estimate: the true effect is likely to be substantially different from the estimate of effect.

Explanations

a. Downgraded by two levels for very serious limitations on bias due to confounding, selection of participants, classification of interventions, and measurement of outcomes domains.
b. Downgraded by two levels for very serious limitations on bias due to confounding, selection of participants, classification of interventions, and measurement of outcomes domains.
c. Downgraded by one level for imprecision.
d. Downgraded by two levels for very serious limitations on bias due to confounding, selection of participants, classification of interventions, and measurement of outcomes domains.
e. Downgraded by two levels for imprecision.
f. Downgraded by two levels for very serious limitations on bias due to confounding, selection of participants, classification of interventions, and measurement of outcomes domains.
g. Downgraded by two levels for imprecision.
h. Downgraded by two levels for imprecision.
